# Supplementary material for: Universal structure of transmission eigenchannels inside opaque media
Source: Nat Commun. 2015 Apr 20;6:6893. doi: 10.1038/ncomms7893 (PMC4411295; doi:10.1038/ncomms7893)
Supplement: Supplementary Information — Supplementary Figures 1-4, Supplementary Notes 1-2 and Supplementary References [file ncomms7893-s1.pdf]

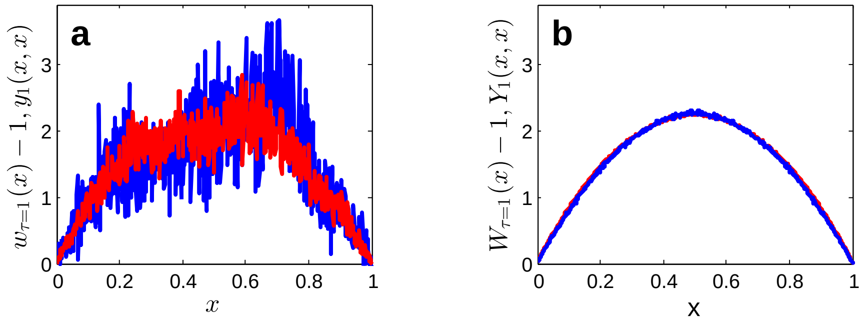

Supplementary Figure 1: Simulation results giving $w_{\tau=1}(x) - 1$ (blue) and $y_1(x, x)$ (red) for a single random sample (a) and their ensemble averages (b). The wave is diffusive in this sample with $N = 35, L/\ell = 5.6$, and $L/\xi = 0.16$.

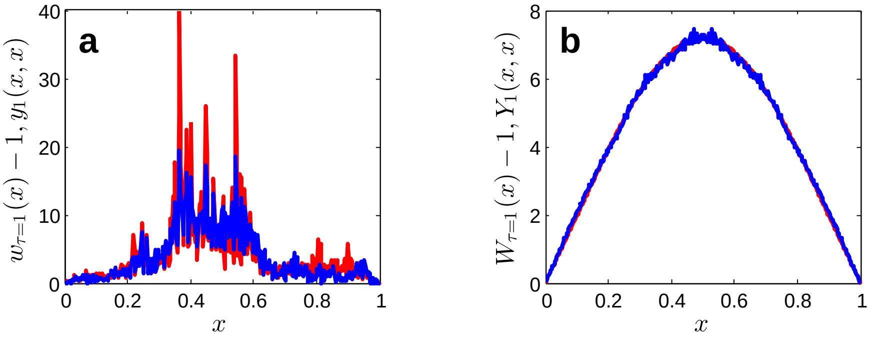

Supplementary Figure 2: The same as Supplementary Fig. 1, but the wave is localized in samples with $N = 11, L/\ell = 18.3$, and $L/\xi = 1.66$.

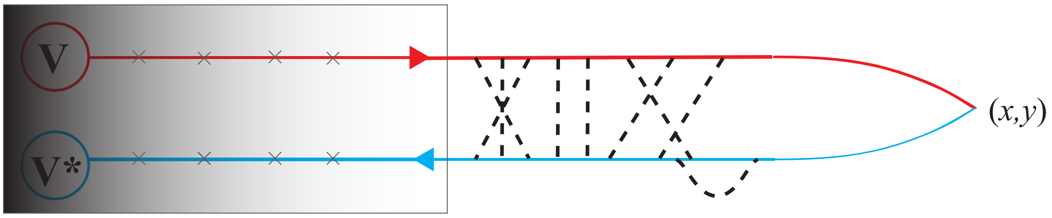

Supplementary Figure 3: A typical diagram representing $\langle|E(x, y)|^2\rangle$. The usual disorder-averaged two-particle Green's function composed of the free propagating lines and the interaction (dashed) line is connected to an opaque box which represents the correlation of $\mathbf{v}(\mathbf{v}^*)$ and dielectric fluctuations (crosses).

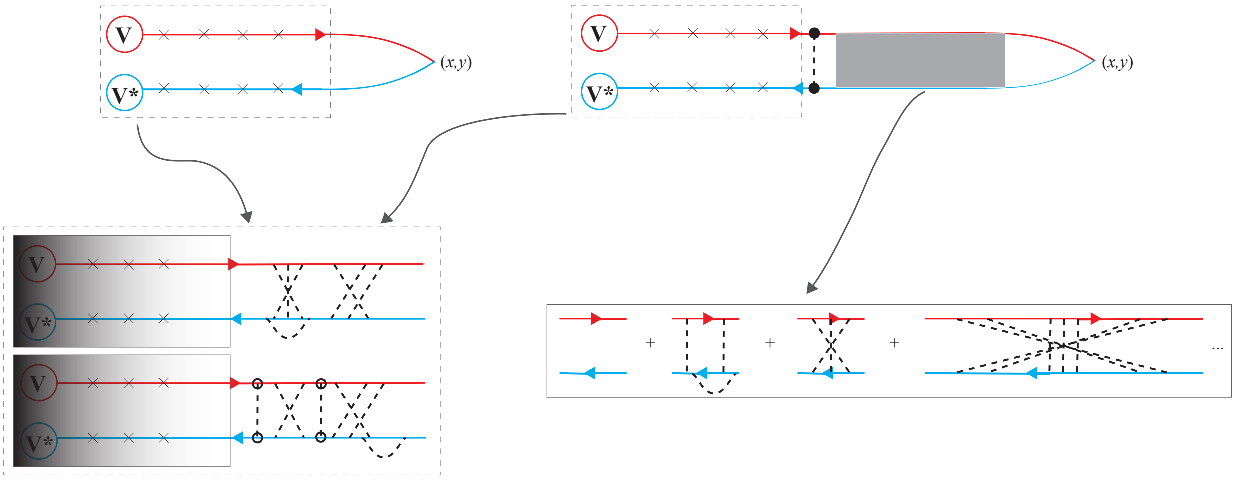

Supplementary Figure 4: Diagrams giving $\langle|E(x,y)|^2\rangle$ for the eigenchannel with specified transmission eigenvalue can be divided into two categories (upper). The first category includes two subclasses with representative examples given in the lower left panel (see the text for explanations). In the second category the diagrams are separated by an interaction (dashed) line into two subdiagrams: the left part consists of diagrams corresponding one-to-one to those in the first category (lower left panel) and the right to those leading to the usual disorder-averaged two-particle Green's function (lower right panel). The solid (empty) circles in the ends of the interaction line stand for that the corresponding integral over the longitudinal coordinate is restricted to (excludes) a layer enclosing (x,y) namely the interval $\mathcal{I}_x$; otherwise there is no restriction on the integral. The line propagating from left (right) to right (left) stands for the Green's function G (G^*).

Supplementary Note 1: Simulations demonstrating the relationship between the energy density profile of the completely transmitting eigenchannel and the return probability to a cross section

Here we demonstrate in simulations the relationship between the profiles of the completely transmitting eigenchannel and the probability for the wave to return to a cross section in a single random configuration of a sample. We denote the energy density profile of the completely transmitting eigenchannel in a single configuration by $w_{\tau=1}(x)$. It is obtained from $G(x,a,x'=0,a')$ in the same way as that described in the main text. The probability density for a wave to return to a cross section in a single configuration at depth x, $y_1(x,x)$, can be evaluated from $N^{-1}\sum_{a,a'}|G(x,a,x'=x,a')|^2$. Here a,a' are the mode indices of the empty waveguide modes. We recall that N is the channel number. For $x=0$, $y_1(x,x)$ must satisfy $y_1(0,0)=0$. Because $\sum_{a,a'}|G(x=0,a,x'=0,a')|^2$ is equal to the sum of the N reflection eigenvalues $1-\tau_n$, this gives $y_1(x,x)=N^{-1}\sum_{a,a'}|G(x,a,x'=x,a')|^2-(1-\sum_n\tau_n/N)$.

The results of simulation results for diffusive and localized waves are shown in Supplementary Figs. 1 and 2, respectively. In each of these figures, panel a shows the profiles of $w_{\tau=1}(x)-1$ (blue) and of $y_1(x,x)$ (red) for a single random sample, while panel b shows the ensemble average for these two functions. These functions differ in a single configuration since the return probability is calculated using the same weight for each modes at x while the enhancement of the energy density in a particular sample at x relates to the actual transverse spatial intensity distribution at x in each sample, which is a random speckle pattern. On average, the energy density in all modes is the same and so the ensemble averages $\langle w_{\tau=1}(x)\rangle-1$ and $\langle y_1(x,x)\rangle$ converge to $W_{\tau=1}(x)-1$ and $Y_1(x,x)$, respectively, as can be seen in Supplementary Figs. 1b and 2b. This is in agreement with the relationships $F_1(x)=Y_1(x,x)$ and $W_{\tau=1}(x)=1+Y_1(x,x)$ established in the main text.

Supplementary Note 2: Diagrammatic theory of the energy density profile of transmission eigenchannels

The energy density profile in a locally 2D sample for a single random configuration and the incoming eigenchannel $\mathbf{v}$ with specific transmission eigenvalue τ, is given by

$$|E(x,y)|^2=\int_0^{L_t}dy'\int_0^{L_t}dy''G(x,y,x'=0,y')G^*(x,y,x'=0,y'')\mathbf{v}(y')\mathbf{v}^*(y''). \qquad (1)$$

We expand the Green's function in terms of dielectric fluctuations $k_0^2\delta\varepsilon$, which are represented by crosses in Supplementary Fig. 3, to obtain

$$G=G_0+G_0(k_0^2\delta\varepsilon)G_0+G_0(k_0^2\delta\varepsilon)G_0(k_0^2\delta\varepsilon)G_0+\cdots, \qquad (2)$$

where $G_0 = 1/(-\nabla^2 - k_0^2 + i0)$ is the free Green's function which is represented by propagating lines in Supplementary Fig. 3. Both the incoming eigenchannel $\mathbf{v}(\mathbf{v}^*)$ and Green's function $G(G^*)$ change with the disorder configuration. Consider the diagrammatic representation of the disorder average of energy density, denoted by $\langle |E(x,y)|^2\rangle$. We find that it must have the general structure shown in Supplementary Fig. 3. On the left of the diagram is an opaque box which represents the correlation of $\mathbf{v}(\mathbf{v}^*)$ and dielectric fluctuations. Connected to this complicated diagrammatic element is the usual disorder-averaged two-particle Green's function, which is composed of the free propagating lines representing $G_0(G_0^*)$ and the interaction (dashed) line. The interaction is introduced by the spatial correlation of dielectric fluctuations, $k_0^4\langle\delta\varepsilon(r)\delta\varepsilon(r')\rangle = \Delta\delta(r-r')$, with Δ being the disorder strength.

All of these diagrams can be divided into two categories (Supplementary Fig. 4). The first category includes two subclasses (upper left): (i) There are no isolated (i.e., not crossed by others interaction lines) connecting upper and lower lines (top of the lower left panel); (ii) Such interaction lines exist, but the integral over the corresponding longitudinal coordinate excludes the regime of $\mathcal{I}_x \equiv (x - \frac{1}{2}v_+\tau_{\rm tr}, x + \frac{1}{2}v_+\tau_{\rm tr})$ namely the layer enclosing the observation point, (x,y), of thickness $v_+\tau_{\rm tr}$, which is represented by dashed lines with empty circles at the ends (bottom of the lower left panel). Here $\tau_{\rm tr}$ is the transport mean free time. In the second category (upper right), a unique interaction line connects upper and lower lines so that the diagram separates into two parts and, moreover, the integral over the corresponding longitudinal coordinate is restricted to the regime of $\mathcal{I}_x$ which is represented by the dashed line with solid circles at the ends.

We first sum all the diagrams in the second category (Supplementary Fig. 4, upper right). This gives

$$\Delta\int_{\mathcal{I}_x} dx'' \int dy'' \left(v_+^{-1}\tilde{S}_\tau(x'',y'')\right)\langle G(x,y,x'',y'')G^*(x,y,x'',y'')\rangle. \tag{3}$$

Here $v_+^{-1}\tilde{S}_\tau(x'',y'')$, represented by a dashed box, accounts for the part on the left of the dashed line whose detailed structure is given in the lower left panel of Supplementary Fig. 4, and the disorder average of the two-particle Green's function, $\langle G(x,y,x'',y'')G^*(x,y,x'',y'')\rangle$, for the part on the right of the dashed line whose detailed structure is given in the lower right panel. Both quantities vary in their arguments over a scale much greater than the transport mean free path $\ell = \mathcal{O}(v_+\tau_{\rm tr})$. It is important to note that only the former, namely, $\tilde{S}_\tau(x'',y'')$, depends on the incoming eigenchannel. In other words, only this part depends on τ. We integrate Eq. (3) over the transverse coordinate y to obtain

$$\begin{aligned} I_\tau(x) &\equiv \Delta\int dy\int_{\mathcal{I}_x} dx''\int dy''\left(v_+^{-1}\tilde{S}_\tau(x'',y'')\right)\langle G(x,y,x'',y'')G^*(x,y,x'',y'')\rangle \\ &\approx \Delta\int dy\int_{\mathcal{I}_x} dx''\int dy''\left(v_+^{-1}\tilde{S}_\tau(x'',y'')\right)\langle G(x,y,x''=x,y''=0)G^*(x,y,x''=x,y''=0)\rangle \\ &= \Delta\left(\tau_{\rm tr}\int dy\tilde{S}_\tau(x,y)\right)\int dy\langle G(x,y,x,0)G^*(x,y,x,0)\rangle. \end{aligned} \tag{4}$$

The correlation function, $\int dy\langle G(x,y,x,0)G^*(x,y,x,0)\rangle$, was previously calculated in the quasi-1D geometry [1] by using the supersymmetry field theory for waves in open scattering media [2]. Substituting the result obtained there into Eq. (4) gives

$$I_\tau(x) = \left(v_+^{-1}\int dy\tilde{S}_\tau(x,y)\right)Y_1(x,x), \tag{5}$$

where $Y_1(x,x)$ is given in Eq. (2) of the main text.

For the diagram in the second category, the dashed box consists of diagrams corresponding one-to-one to those in the first category. Therefore, the sum of the latter is $v_+^{-1}\tilde{S}_\tau(x,y)$. The total energy density integrated over the transverse coordinate is the sum of $v_+^{-1}\int dy\tilde{S}_\tau(x,y)$ and $I_\tau(x)$, i.e.,

$$\int dy\langle|E(x,y)|^2\rangle = v_+^{-1}\int dy\tilde{S}_\tau(x,y) + I_\tau(x) = \left(v_+^{-1}\int dy\tilde{S}_\tau(x,y)\right)(1+Y_1(x,x)). \tag{6}$$

The first factor in the second equality depends on τ but the second factor does not. This justifies the factorization of $W_\tau(x)$, namely Eq. (3) in the main text, with

$$S_\tau(x) = \int dy\tilde{S}_\tau(x,y). \tag{7}$$

As such, the diagrammatic technique gives the precise meaning of the source strength $S_\tau(x)$ which is the sum of all the diagrams in the first category integrated over the transverse coordinate.

---

[1] Tian, C.-S., Cheung, S.-K. & Zhang, Z.-Q. Local diffusion theory for localized waves in open media. Phys. Rev. Lett. **105**, 263905 (2010).
[2] Tian, C. Supersymmetric field theory of local light diffusion in semi-infinite media. Phys. Rev. B **77**, 064205 (2008).
